# Exploring the Patterns of Acupuncture on Mild Cognitive Impairment Patients Using Regional Homogeneity

**DOI:** 10.1371/journal.pone.0099335

**Published:** 2014-06-26

**Authors:** Zhenyu Liu, Wenjuan Wei, Lijun Bai, Ruwei Dai, Youbo You, Shangjie Chen, Jie Tian

**Affiliations:** 1 Key Laboratory of Molecular Imaging and Functional Imaging, Institute of Automation, Chinese Academy of Sciences, Beijing, China; 2 Department of Acupuncture and Massage, Bao’an Hospital, Southern Medical University, Shenzhen, China; 3 Life Sciences Research Center, School of Life Sciences and Technology, Xidian University, Xi’an, Shaanxi, China; University of Pennsylvania, United States of America

## Abstract

**Purpose:**

To investigate the different responses to acupuncture in MCI patients and age-matched healthy subjects reflected by the Regional Homogeneity (ReHo) indices.

**Methods:**

The experiment was performed at the acupoint KI3 in 12 MCI patients and 12 healthy controls, respectively. A novel non-repeated event-related (NRER) fMRI design paradigm was applied to separately detect neural activities related to different stages of acupuncture (pre-acupuncture resting state, needling manipulation and post-acupuncture resting state). ReHo values were calculated for MCI patients and healthy controls in pre- and post-acupuncture resting state. Then, a two-way ANCOVA with repeated measures with post-hoc two sample t-tests was performed to explore the different responses to acupuncture in the two groups.

**Results:**

The ANCOVA revealed a significant main effect of group, but no significant main effect of acupuncture and interactions between group and acupuncture. During the pre-acupuncture resting state, ReHo values increased in the precentral gyrus (PCG), superior frontal gyrus (SFG), and insula (INS) and decreased mainly in middle temporal gyrus (MTG), parahippocampal (PHIP) and cingulate cortex in MCI patients compared with healthy controls. Furthermore, we found that the regions including precuneus (PCUN), and cingulate cortex showed increased ReHo values for MCI patients following acupuncture. For healthy controls, the medial frontal gyrus, PCG, anterior cingulate cortex (ACC) and INS showed enhanced ReHo values following acupuncture. During the post-acupuncture resting state, MCI patients showed increased ReHo values mainly in the MTG, superior parietal lobule (SPL), middle frontal gyrus (MFG), supramarginal (SMG), and PCG, and decreased ReHo values mainly in the frontal regions, PHIP, and posterior cingulated cortex (PCC) compared to healthy controls.

**Conclusion:**

Though we found some ReHo changes between MCI patients and healthy controls, the two-way ANCOVA results showed no significant effects after multiple corrections. Further study is needed to reveal the real acupuncture effects on MCI patients.

## Introduction

Alzheimer’s disease (AD), the most common form of dementia, is characterized by significant impairments in multiple cognitive domains including memory, attention, reasoning, language and executive-functions. There is currently little effective disease-modifying treatment, and many potential treatments being tested may have significant side-effects. Given that pathological changes begin well before the appearance of clinical symptoms [1], earlier interventions could improve disease prognosis. As an intermediate state between normal aging and dementia, the most prominent feature of mild cognitive impairment (MCI) is an isolated mild decline in memory, whereas other cognitive functions remain intact [2]. MCI patients would turn into AD at a high rate of approximately 10% to 15% per year [3]. MCI has become a hot topic of dementia researchers in recent years. The increased risk for the elderly that suffer from MCI to progress to AD makes it an appropriate condition for investigation.

The use of acupuncture as a complementary therapeutic method for treating a variety of neurologic diseases, including MCI, AD and dementia, is popular in certain parts of the world [4]. Previous study suggested that acupuncture had significant therapeutic effects and well tolerated in ameliorate the key clinical symptoms of vascular dementia [5]. Luijpen et al. [6] explored the effects of transcutaneous electrical nerve stimulation on self-efficacy and mood in MCI patients by measuring four outcomes including a Dutch translation of the General Self-Efficacy Scale (Algemene Competentie Schaal), the Groninger Activity Restriction Scale, the Philadelphia Geriatric Center Morale Scale, and the Geriatric Depression Scale. The results indicated that the transcutaneous electrical nerve stimulation could improve the outcomes compared with placebo. In spite of its public acceptance, the neural mechanism underlying is still elusive. In the past decades, noninvasive fMRI technique has provided new insights into the central physiological function involving acupuncture. Neuroimaging studies of acupuncture have indicated that primary acupuncture effects are mediated by the central nervous system [7]–[20]. Previous neuroimaging studies [7]–[11] on acupuncture at commonly used acupoints have demonstrated significant modulatory effects involving widespread cerebrocerebellar brain regions including the limbic, hippocampus, somatosensory cortices, hypothalamic, insula and brainstem neural nuclei. However, the majority of these studies were performed on healthy subjects. It is generally agreed that acupuncture plays a homeostatic role and thus may have a greater effect on patients with a pathological imbalance, compared with healthy controls [21], [22]. Therefore, exploring the effects of acupuncture on the central nervous system in patients may further help to elucidate its mechanism. One previous study adopts fMRI to explore the acupuncture effects in 26 AD patients [23]. The results showed that acupuncture could activate the temporal lobe (such as the hippocampus, and insula), some regions of the parietal lobe and cerebellum in AD patients. The regions activated by acupuncture are consistent with impaired brain areas in AD patients. Meanwhile, these regions are also closely correlated with the cognitive function (memory, reasoning, language, executive, and etc.). This study provides preliminary neurophysiological evidence for the potential efficacy effect of acupuncture on AD. Another recent study [24] investigating the acupuncture effects on the functional connectivity in MCI patients using deep and superficial acupuncture found significantly increased correlations related with the temporal regions following deep acupuncture. However, few fMRI studies focused on the modulatory effects of acupuncture on brain regions in MCI patients. Hence, imaging the regions mainly affected in MCI patients may help to unravel the mechanisms how acupuncture achieves its therapeutic effects.

According to the theory of the Traditional Chinese Medicine, acupuncture can induce long-lasting effects even after the needling manipulation being terminated [25]. Recently, several studies demonstrated the existence of various function-guided brain networks even after the needling manipulation, which underlay the prolonged effects of acupuncture [12], [15], [16], and the block design may be not fit for the acupuncture study. Therefore, the sustained effects of acupuncture should be taken into account for which the actual effects of acupuncture can be appropriately studied. In the present study, we adopted the newly non-repeated event-related (NRER) fMRI design [13]. This new design could be used to explore the sustained effect of acupuncture (the modulatory patterns during the resting state following acupuncture). In the present study, we used the NRER design to investigate whether the effects of acupuncture on ReHo are different for MCI patients and healthy controls during the resting state following acupuncture.

Regional Homogeneity (ReHo) [26], evaluating the similarity between the time series of a given voxel and its nearest neighbors by calculating Kendall’s coefficient of concordance (KCC) [27], can effectively evaluate the resting-state brain activity. On the other hand, ReHo is a reliable method with its test-retest reliability established recently by Zuo et al [28]. Therefore, ReHo can rapidly map the level of regional activity of every voxel across the whole brain of individuals [29]. A previous study [30] found that in amnestic MCI patients, the ReHo indices were decreased in regions that included the PCC, the right anterior cingulate cortex (ACC), the right inferior frontal regions, the right STG and the bilateral cuneus, and were increased in the right IPL, the right fusiform gyrus and the bilateral putamen. In a recent study [31], differences in the ReHo indices between MCI and healthy controls were mainly found in the medial prefrontal cortex (mPFC), bilateral PCC and left IPL. By calculating ReHo indices, we may evaluate the different responses to acupuncture in MCI patients during the following acupuncture resting state. Studying ReHo indices provides us a new way to exploring the mechanisms of acupuncture and will be helpful for a better understanding of acupuncture effects on MCI.

In the present study, we sought to investigate the different responses of MCI patients and healthy controls to acupuncture at the same acupoint reflected by the ReHo indices. The fMRI experiment was performed at an acupoint KI3. We first performed a two-way ANCOVA with repeated measures (two factors: group, acupuncture; repeated factor: acupuncture; covariates: gender, head motion) to test the main effect of group and acupuncture, and the interaction of the two factors. Then we identified regions showing abnormal ReHo indices in MCI group compared with the HC during the resting state. Subsequently, we tested the effects of acupuncture on MCI patients and healthy controls, respectively. Finally, we made the comparison between MCI patients and healthy controls to test whether there were any specific modulatory patterns during post-acupuncture resting state. We hypothesized that the specific effects of acupuncture on MCI patients may be reflected by the ReHo indices.

## Materials and Methods

All research procedures were approved by the Bao’an People’s Hospital Subcommittee on human studies and conducted in accordance with the Declaration of Helsinki.

### Subjects

12 MCI patients and 12 age-matched healthy control subjects were included in this study (see Table 1 for subjects’ characteristics). MCI patients were recruited at the rehabilitation department of the Bao’an People’s Hospital of Shenzhen. MCI patients were diagnosed using criteria for amnestic MCI [32], with Mini-Mental State Examination (MMSE) scores between 25 and 27 [33], and Clinical Dementia Rating (CDR) scale scores of 0.5 [34]. Healthy control subjects were recruited from a community. All subjects were right-handed according to the Edinburgh Handedness Inventory [35]. In addition, all subjects were acupuncture naive as several previous studies [17], [36], [37] did, because previous experience of acupuncture is believed to affect people’s expectation of future treatments. Using non-naïve subjects may account for placebo effects in acupuncture study [38]. Subjects were excluded if they had any significant medical, neurological, or psychiatric illness, or if they were taking medication or other substances known to influence cerebral function. After given a complete description of the study, all subjects signed the informed consent form. All protocols were approved by the Bao’an People’s Hospital Subcommittee on human studies.

**Table 1:** Subject characteristics.

	Patients	Controls
N	12	12
Age (mean ± SD)	59.3±3.3	60.6±5.8
Sex(M/F)	1/11	4/8
Education (year)	10.5±1.81	10.6±2.06
MMSE score * (mean ± SD)	26.4±0.9	29.8±0.4
CDR	0.5	0

MMSE: Mini-Mental State Examination, CDR: Clinical Dementia Rating.

* *p*<0.0001 (two sample two tailed *t* test).

### Experimental Paradigm

**Figure 1:**
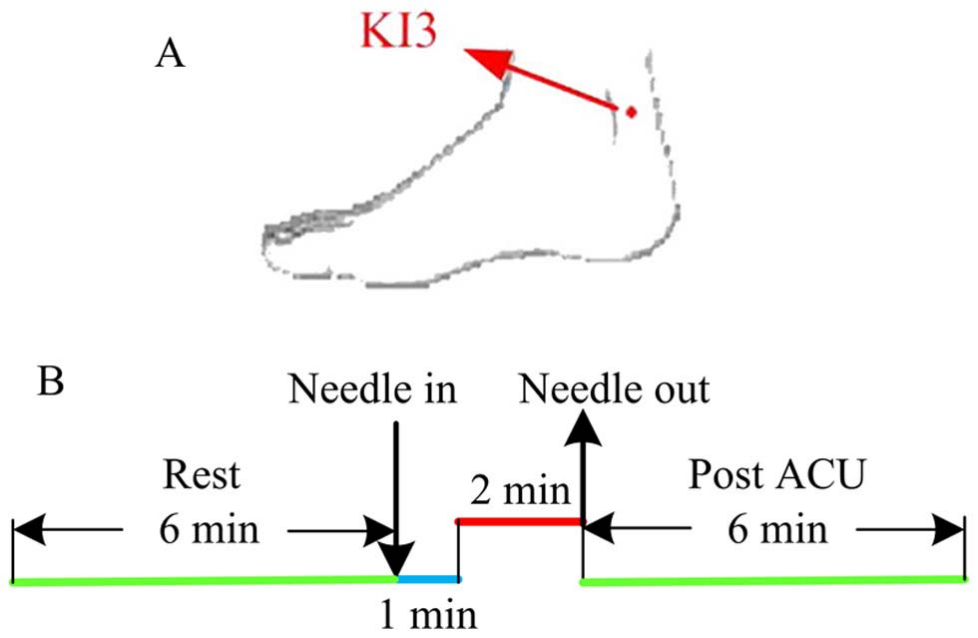
Experimental paradigm. A: The location of the acupoint used in the experiment. B: The paradigm of the experiment: the resting state run lasted for 6 minutes; acupuncture run totally lasted for 9.0 minutes.

In this study, we adopted a new experimental paradigm, namely the NRER fMRI design to investigate the prolonged effects after acupuncture administration [13]. For each group, the experiment consisted of two functional runs. For a baseline control, a resting state (REST) scan was conducted for 6 minutes without any stimulation (Fig. 1A). We then employed the new experimental paradigm, in which acupuncture was conducted and only one stimulation period was given (Fig. 1B). For acupuncture run, an acupuncture needle was inserted from the beginning, and after resting for 1 min, the needle was manipulated for 2 minutes; then, another resting state scan was conducted for 6 minutes without any stimulation. All participants were asked to keep their eyes closed and remain relaxed without engaging in any mental tasks. According to participants’ reports after the scanning, they affirmed keeping awake during the whole process. At the end of acupuncture scan, the subjects adopted a 10-point scale to self-rate the intensities about the deqi sensations they had felt during the stimulation (0 =  no sensation, 1–3 =  mild, 4–6 =  moderate, 7–8 =  strong, 9 =  severe and 10 =  unbearable sensation) [39].

Acupuncture was performed at an acupoint KI3 (Taixi, located in a depression between medial malleolus and heel tendon) on the right leg. This is one of the most frequently used acupoints and proved to have various efficacies in the treatments of dementia [23]. Acupuncture stimulation was delivered using a sterile disposable 38 gauge stainless steel acupuncture needle, 0.2 mm in diameter and 40 mm in length. The needle was inserted vertically to a depth of 1–2 cm, and administration was delivered by a balanced “tonifying and reducing” technique [7]. Stimulation consisted of rotating the needle clockwise and counterclockwise for 1 min at a rate of 60 times per min. The procedure was performed by the same experienced and licensed acupuncturist on all participants.

### Data Acquisition and Preprocessing

Magnetic resonance imaging data were acquired using a 3.0 Tesla Signa (GE) MR scanner. Head movements were prevented by a custom-built head holder. The images were parallel to the AC-PC line and covered the whole brain. Thirty axial slices were obtained using a T2*-weighted single-shot, gradient-recalled echo planar imaging sequence (FOV = 220 mm×220 mm, matrix = 64×64, thickness = 4 mm, TR = 2000 ms, TE = 30 ms, flip angle = 77°). After the functional run, high-resolution structural information on each subject was also acquired using 3D MRI sequences with a voxel size of 1 mm3 for anatomical localization (TR = 2.1 s, TE = 4.6 ms, matrix = 256×256, FOV = 230 mm×230 mm, flip angle = 8°, slice thickness = 1 mm).

For REST run, the data were preprocessed by removing the first 5 time points to eliminate nonequilibrium effects of magnetization. For acupuncture run, only the datasets after manipulation were selected (labeled by green-color in Fig. 1, total of 180 time points, the same time points as in REST run), and the first 5 time points were discarded in order to obtain a stable resting state. The remaining time points were used for following analysis. All of data preprocessing procedures were performed in Statistical Parametric Mapping 5 (SPM5) (http://www.fil.ion.ucl.ac.uk/spm). All the remaining volumes were firstly realigned to correct for head motions using the least-squares minimization. The images were corrected for the acquisition delay between slices, aligned to the first image of each session for motion correction, and spatially normalized to standard MNI template in SPM5 [40]. No subjects had head motions exceeding 1 mm movement or 1° rotation in any direction. Finally, A band-pass filter (0.01 Hz<f<0.08 Hz) was applied to remove physiological and high frequency noise [41].

### Data Processing and Statistical Analysis

Kendall’s coefficient concordance (KCC) [27] was used to evaluate regional homogeneity [26], which was performed using the Resting-State fMRI Data Analysis Toolkit (REST, by SONG Xiao-Wei et al. [42], http://www.restfmri.net). Individual ReHo maps were generated by assigning each voxel a value corresponding to the KCC of its time series with its nearest 26 neighboring voxels [26]. A whole brain mask was used to remove non-brain tissues. Only the voxels within the mask were further analyzed. The individual ReHo maps were standardized by their own mean KCC within the mask [43], [44]. In this step, KCC value of each voxel was divided by the mean KCC within the mask in each individual ReHo map. To test if there were any changes of global mean ReHo across groups as well as acupuncture, we performed a two way ANCOVA with repeated measures (two factors: group, acupuncture; repeated factor: acupuncture; covariates: gender, head motion). Then, a Gaussian kernel of 4 mm full width at half-maximum (FWHM) [45]–[47] was used to smooth the images for the aim to reduce noise and residual differences.

Statistical analysis was using SPM5 (http://www.fil.ion.ucl.ac.uk/spm). A one-sample t test (p<0.05, false discovery rate (FDR) correction) was performed to extract the ReHo results across the subjects within each group for both conditions. We first performed a two-way ANCOVA with repeated measures in the present study to test the main effect of group and acupuncture, and the interaction of the two factors. Then we performed two-sample t-test to test the differences between HC and MCI patients in both rest condition and post acupuncture resting state. We also compared the ReHo results between post-acupuncture and rest by performing paired t-test in each group.

## Results

### Psychophysical Response

**Figure 2:**
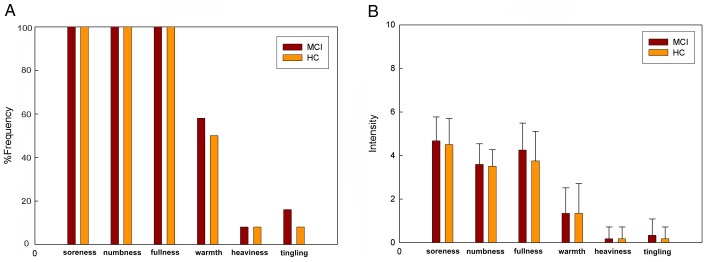
Averaged psychophysical response (N = 12) in MCI and HC groups. A: The percentage of subjects who reported having experienced the given sensation (at least one subject experienced the six sensations listed). B: The intensity of reported sensations measured by an average score (with standard error bars) on a scale from 0 denoting no sensation to 10 denoting an unbearable sensation.

The prevalence of subjective “deqi” sensations was expressed as the percentage of individuals in the group that reported the given sensations (Fig. 2A). For both conditions, no statistically significant changes were found with regard to the prevalence of the listed sensations between MCI and HC (paired t-test, P>0.05). However, differences did exist with respect to the type of sensations. Warmth (MCI: 58%, HC: 50%), tingling (MCI: 16%, HC: 8%) were found greater in MCI group. The intensity of sensations was expressed as the average score ± SE (Fig. 2B). The averaged intensities were approximately similar in MCI and HC groups (paired t-test, P>0.05). Considering a little difference in psychophysical response between MCI and HC, the neuroimaging findings were likely not the results of differences induced by the sensations.

### ReHo Results of each Condition in the Two Groups

**Figure 3:**
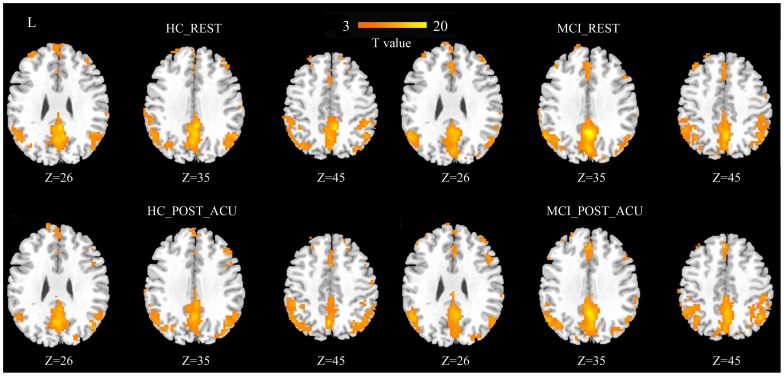
Results of regional homogeneity (ReHo) shown as a Kendall’s coefficient of concordance (KCC) map across all subjects for MCI patients and healthy controls during resting state and post-acupuncture resting state (p<0.05, FDR correction).

ReHo results across all subjects of the two groups during the two conditions are shown in Figure 3 (p<0.05, FDR correction). The major regions of DMN exhibited significant higher ReHo values than other brain regions during the resting state (Fig. 3 A, B), i.e. the MTL, PCC, mPFC, and IPL.

### Results of Two-way ANCOVA

We found no changes of global mean ReHo values across groups as well as acupuncture using the two way ANCOVA with repeated measures (P<0.05).

**Figure 4:**
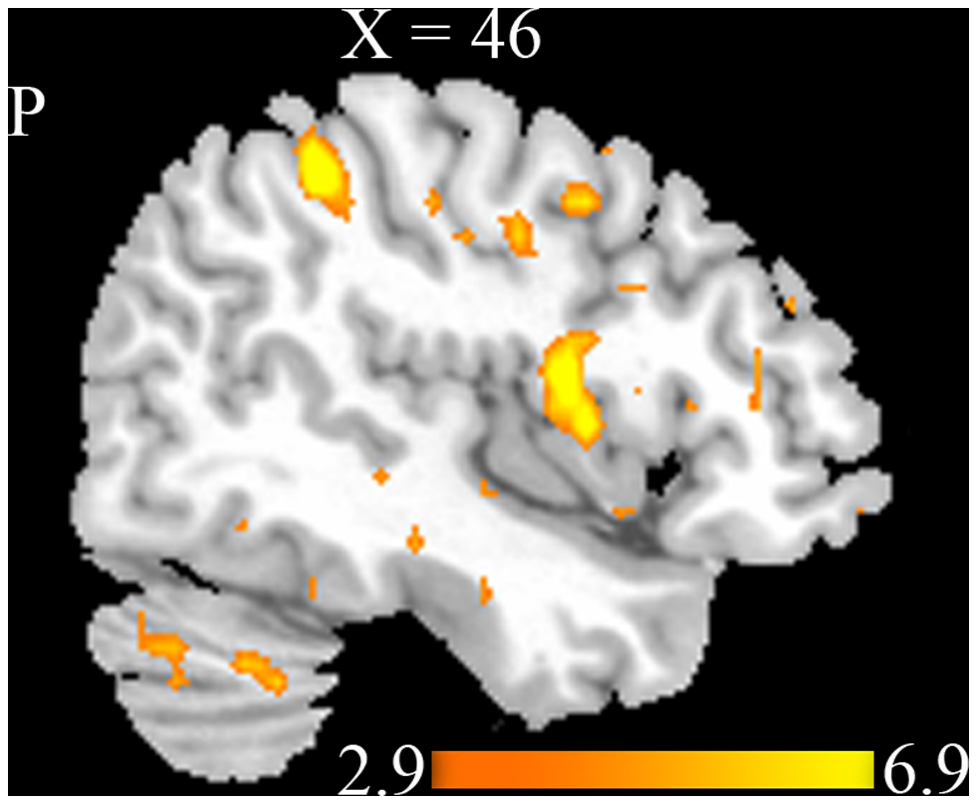
Brain areas showed significant main effect of group at p<0.001 (uncorrected, cluster size >30 voxels) with increased ReHo values in MCI patients compared with normal controls. Warm colors indicate the significantly increased ReHo values.

We performed a two-way ANCOVA with repeated measures to test the main effect of group and acupuncture, and the interaction of the two factors. The analysis revealed a significant main effect of group at p<0.001 (uncorrected, 30 voxels) with the regions including the PCG, SPL, INS, STG, and Inferior Temporal Gyrus (ITG) showing increased ReHo values in MCI patients (Fig. 4 and Table 2 ). The results suggested that the brain spontaneous activity differs between MCI patients and healthy controls during resting state and post acupuncture resting state. We found no significant main effect of acupuncture and interaction of the two factors at p<0.001 (uncorrected, 30 voxels) from the two-way ANCOVA. Then, we performed post hoc two sample t-tests to characterize the statistical differences between MCI patients and healthy controls before and after acupuncture respectively.

**Table 2:** Brain areas with significant main effect of group at *P*<0.001 (uncorrected, 30 voxels).

Brain regions	BA	Hemisphere	MNI			Maximal Z-score	Volume (voxels)
			*x*	*y*	*z*		
Precentral Gyrus	6	L	−39	−12	66	6.44	32
Superior Parietal Lobule	7	L	−24	−63	57	5.98	73
Insula	13	L	−30	−24	21	5.92	36
Superior Temporal Gyrus	39	R	39	−51	27	5.58	111
Inferior Temporal Gyrus	20	L	−51	0	−36	5.52	72

### Comparisons for MCI vs. HC in REST

**Figure 5:**
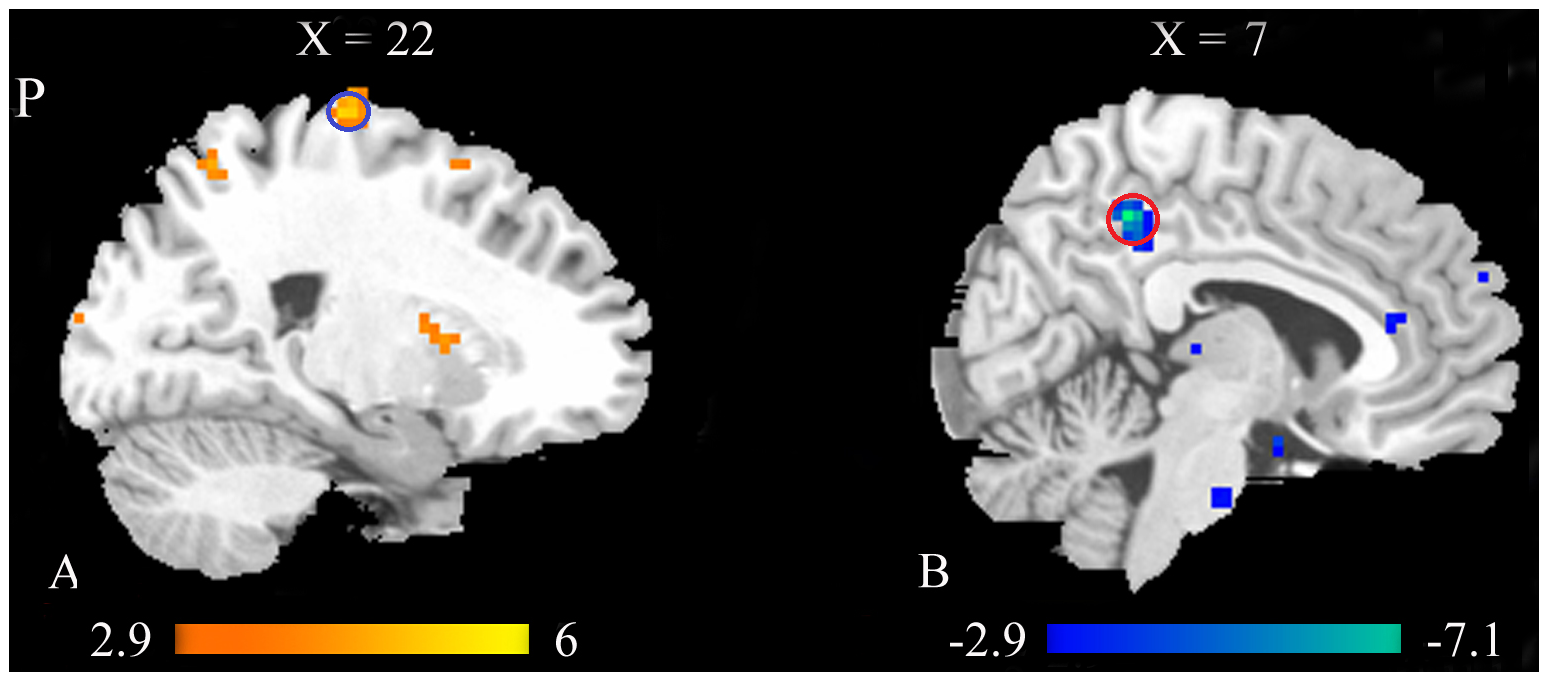
Brain areas with significant different ReHo values between MCI and HC groups during the resting state are shown. Warm and cool colors indicate the significantly increased and decreased ReHo values in MCI patients compared with healthy controls at P<0.01 (Alphasim corrected, p<0.01, 30 voxels). (Abbreviation: PCG- Precentral Gyrus).

We first made a comparison between MCI patients and healthy controls to find the regions showing abnormal ReHo values in MCI patients compared with healthy controls during the resting state (Fig. 5 and Table 3). The results of two sample t test revealed that MCI patients showed significantly increased ReHo values at *P*<0.01 (Alphasim corrected, *p*<0.01, 30 voxels) in the regions including the PCG, SFG, cuneus and INS. Meanwhile, MCI patients showed significantly decreased ReHo values at *P*<0.01 (Alphasim corrected, *p*<0.01, 30 voxels) mainly in the MTG, PHIP and cingulate cortex.

**Table 3:** Brain areas with significant different ReHo values between MCI patients and healthy controls during the resting state at *P*<0.01 (Alphasim corrected, *p*<0.01, 30 voxels).

Brain regions	BA	Hemisphere	MNI			Maximal Z-score	Volume (voxels)
			*x*	*y*	*z*		
Precentral Gyrus	6	L	−21	−24	72	5.44	86
Superior Frontal Gyrus	6	–	0	2	51	4.28	30
Cuneus	18	–	0	−99	0	4.66	35
Insula	13	R	33	9	15	3.74	31
Cingulate Gyrus	31	L	−6	−48	42	−7.11	62
ParaHippocampal	20	R	33	−15	−39	−4.71	37
	37	L	−30	−45	−15	−5.27	39
Middle Temporal Gyrus	21	L	−51	0	−33	−4.82	66

### Comparisons for Acupuncture Effects in each Group

**Figure 6:**
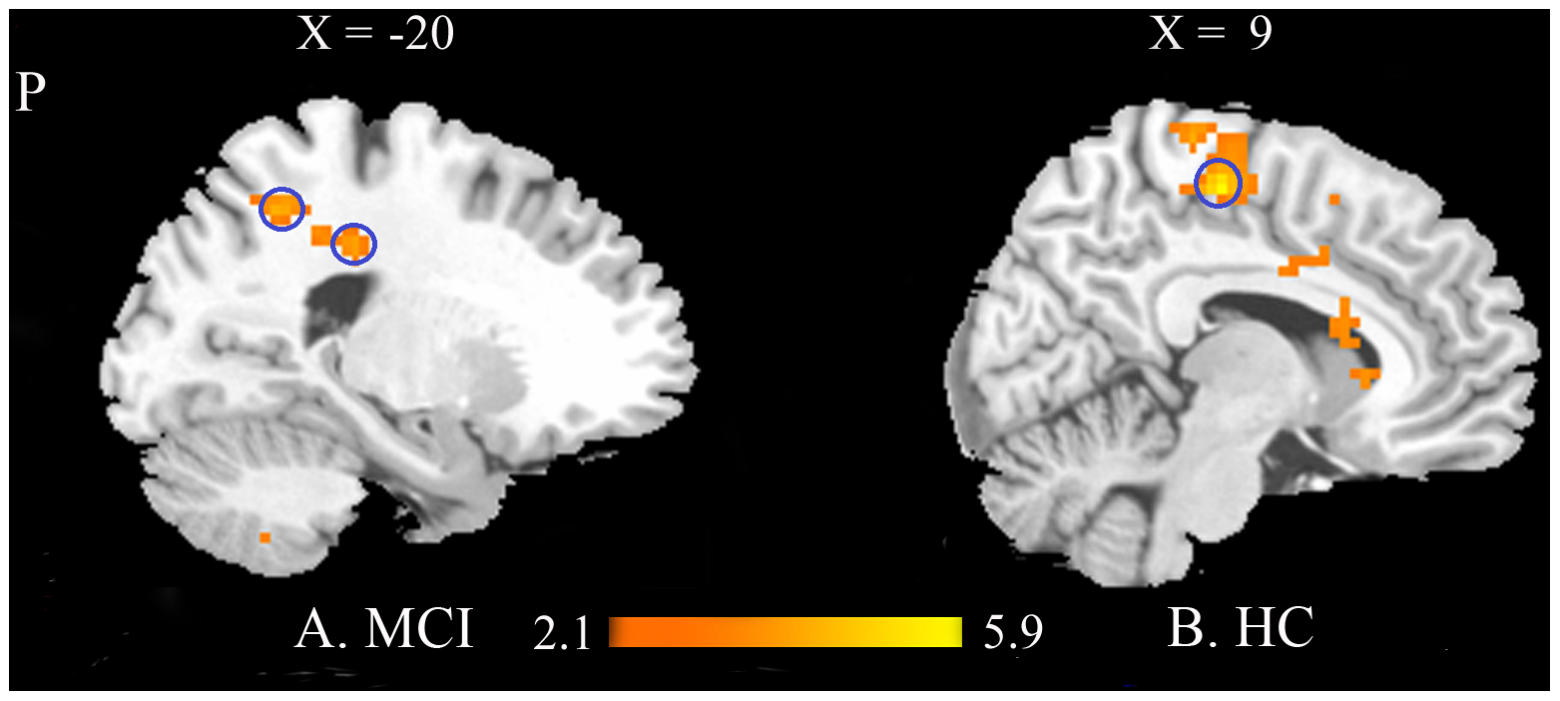
Brain areas with significant different ReHo values following acupuncture compared with the resting state in MCI (A) and HC (B) groups. Warm colors indicate the significantly increased ReHo values in the two groups following acupuncture at *P*<0.01 (Alphasim corrected, *p*<0.01, 30 voxels). (Abbreviation: MFG- Medial Frontal Gyrus).

Then, we performed comparisons in both MCI and HC groups between post acupuncture condition and the resting state to explore the effects of acupuncture on MCI patients and healthy controls, respectively (Fig. 6 and Table 4, 5). For MCI patients, precuneus, and cingulate cortex showed significantly increased ReHo values at *P*<0.01 (Alphasim corrected, *p*<0.01, 30 voxels). While for HC group, significantly increased ReHo values were mainly found in the regions including MFG, PCG, ACC, and INS at *P*<0.01 (Alphasim corrected, *p*<0.01, 30 voxels). No brain regions with significantly decreased ReHo values were found in both groups at *P*<0.01 (Alphasim corrected, *p*<0.01, 30 voxels).

**Table 4:** Brain areas with significant different ReHo values in the MCI patients after acupuncture at *P*<0.01 (Alphasim corrected, *p*<0.01, 30 voxels).

Brain regions	BA	Hemisphere	MNI			Maximal Z-score	Volume (voxels)
			*x*	*y*	*z*		
Cingulate Gyrus	31	R	21	−33	36	3.31	42
Precuneus	7	R	21	−57	45	3.84	36

**Table 5:** Brain areas with significant different ReHo values in the healthy controls after acupuncture at *P*<0.01 (Alphasim corrected, *p*<0.01, 30 voxels).

Brain regions	BA	Hemisphere	MNI			Maximal Z-score	Volume (voxels)
			*x*	*y*	*z*		
Medial Frontal Gyrus	6	L	−6	−24	54	5.85	114
	32	R	12	12	48	3.23	40
Precentral Gyrus	6	R	54	−9	54	3.08	49
Anterior Cingulate Cortex	25	L	−3	18	0	3.97	35
Insula	13	R	34	−15	16	3.48	33

### Comparisons for MCI vs. HC for Acupuncture Condition

**Figure 7:**
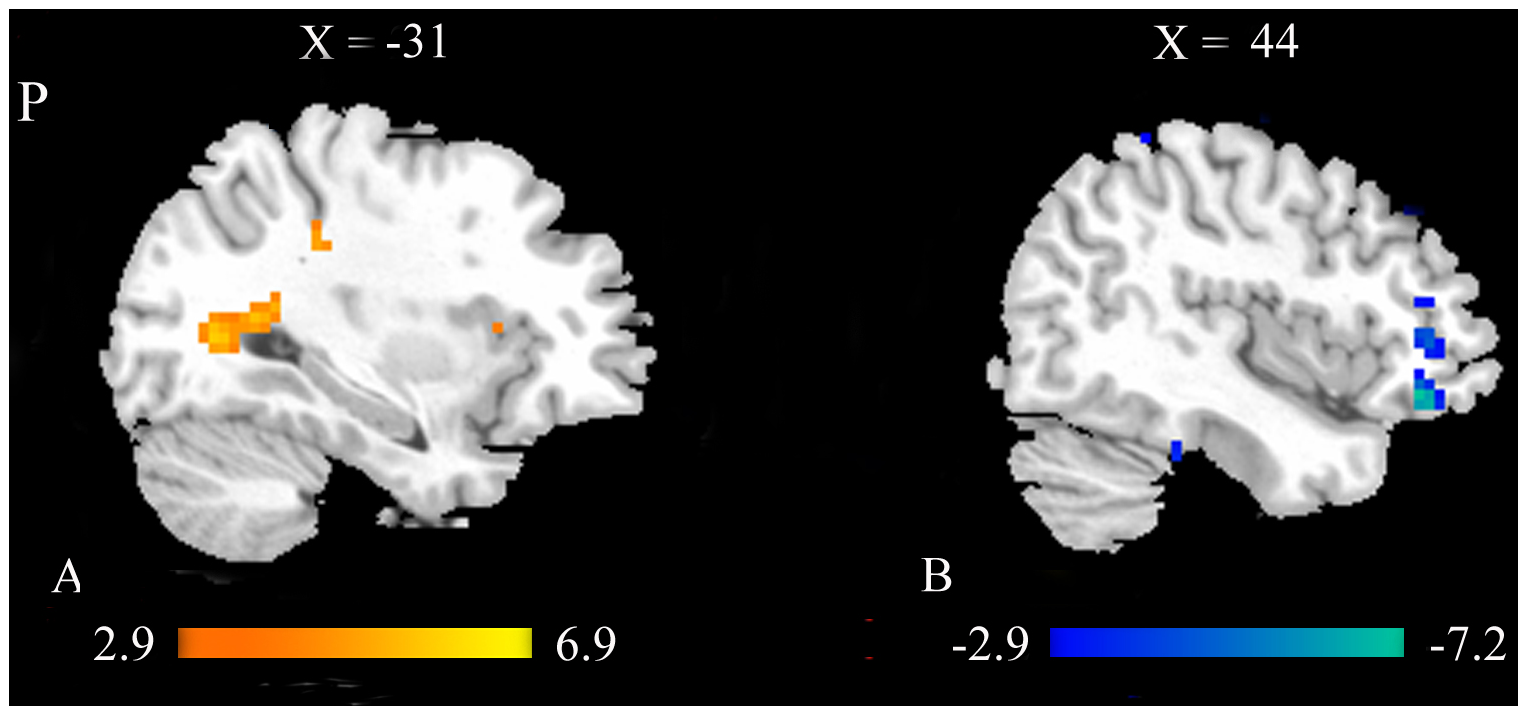
Brain areas with significant different ReHo values between MCI and HC groups during the resting state following acupuncture. Warm and cool colors respectively indicate the significantly increased (A) and decreased (B) ReHo values in MCI patients compared with healthy controls at *P*<0.01 (Alphasim corrected, *p*<0.01, 30 voxels). (Abbreviation: MTG- Middle Temporal Gyrus, MFG- Middle Frontal Gyrus).

Finally, we made a comparison between MCI group and HC group to investigate the different effects of acupuncture on MCI patients and healthy controls (Fig. 7 and Table 6). The results of two sample t test demonstrated that the regions including MTG, SPL, MFG, SMG, cuneus and PCG showed significantly increased ReHo values in MCI patients compared with healthy controls at *P*<0.01 (Alphasim corrected, *p*<0.01, 30 voxels). Meanwhile, significantly decreased ReHo values in MCI patients were found in the regions including the frontal regions (IFG, MFG, and SFG), fusiform gyrus, PHIP, ITG and PCC at *P*<0.01 (Alphasim corrected, *p*<0.01, 30 voxels).

**Table 6:** Brain areas with significant different ReHo values between healthy controls and MCI patients after acupuncture at *P*<0.01 (Alphasim corrected, *p*<0.01, 30 voxels).

Brain regions	BA	Hemisphere	MNI			Maximal Z-score	Volume (voxels)
			*x*	*y*	*z*		
Middle Temporal Gyrus	39	R	39	−54	6	5.11	95
Superior Parietal Lobule	7	L	−27	−63	57	5.27	32
Supramarginal Gyrus	40	L	−39	−51	30	3.85	40
Middle Frontal Gyrus	46	R	45	39	21	4.71	44
Precentral Gyrus	4	L	−24	−24	75	4.61	36
Cuneus	18	–	0	−99	0	4.54	35
Posterior Cingulate Cortex	30	L	−3	−60	−6	−6.68	68
Middle Frontal Gyrus	47	L	−42	36	0	−4.43	31
Superior Frontal Gyrus	9	R	6	54	30	−3.84	32
Fusiform Gyrus	36	L	−39	−39	−30	−4.05	36
Parahippocampal Gyrus	35	L	−24	−15	−30	−4.76	35
Inferior Temporal Gyrus	21	L	−57	−20	−24	−5.11	49
Inferior Frontal Gyrus	47	L	−39	33	−15	−7.21	47

## Discussion

In the present study, we explored the effects of acupuncture on post-stimulus resting brain of MCI patients by evaluating the ReHo indices. Most resting state fMRI studies adopted functional connectivity to investigate temporal relations between intrinsic fluctuations observed in spatially distinct brain regions. However, functional connectivity provides little local features of spontaneous brain activity observed in specific regions. As a complement of the functional connectivity method, ReHo has been proved to be sufficient to detect regional homogeneity abnormality in the brain during the resting state [44], [46], [47], [48]. ReHo hypothesizes that voxels within a specific functional brain region are more temporally homogeneous when subjects are engaged in a specific condition [26]. Previous studies reported that MCI would result in the abnormal ReHo values in related regions and suggested that this method may be an important factor to improve the understanding of the disease [30], [31]. Therefore, changes of ReHo indices during the post-acupuncture resting state may underlie the mechanisms how acupuncture treats MCI. However, few studies investigated the modulatory of acupuncture on MCI patients using ReHo indices. Another advantage of the present study is the use of NRER design. Previous acupuncture studies generally adopted the multi-block design paradigm, which implicitly presumes the temporal intensity profiles of the certain event conforming to the “on - off” specifications [7], [8], [11]. Due to the sustained effects of acupuncture, the temporal aspects of the blood oxygen level dependent response to acupuncture may violate the assumptions of the block designed estimates [17]. In the present study, we adopted the new experimental paradigm named NRER fMRI design, which has been proved to be more applicable to the acupuncture research proved in several previous studies [13], [18], [19].

During the resting state, we found the ReHo values significantly increased in the PCG, SFG, cuneus and INS in MCI patients compared with healthy controls. Meanwhile, we found decreased ReHo values mainly in the MTG, PHIP and cingulate cortex in MCI patient compared with healthy controls. These regions are implicated in memory encoding and retrieving. The result is compatible with previous studies that indicated the dysfunctional regions in MCI patients [49]–[51].

We evaluated the differences between the ReHo indices of the post acupuncture resting state and the ReHo indices of the resting state in both MCI and HC groups to explore the responses of the central nervous system to acupuncture. Though we detected no significant main effect of acupuncture from the two-way ANCOVA, we found changes in ReHo values after acupuncture in MCI patients and healthy controls. We supposed that the small sample size of subjects and no measure of cognitive outcomes may be the reason why we detected no significant main effect of acupuncture. With a large sample size and cognitive outcomes as covariates in ANCOVA, we may detect more precise results of acupuncture effects. In MCI patients, we found that the regions including precuneus, and cingulate cortex showed increased ReHo values during the post acupuncture resting state. The results suggested that acupuncture may enhance the regional connectivity of the abnormal brain regions in MCI patients. Instead of the regions related to cognitive functions, healthy controls showed increased ReHo values in the regions including the MFG, PCG, ACC, and INS during the post stimulus resting state. These different responses to acupuncture between MCI patients and health controls may be related to the different patterns of acupuncture on the two groups.

In order to find out the different effects of acupuncture between MCI patients and healthy controls, we performed a comparison between MCI patients and healthy controls during the post acupuncture resting state. We found that ReHo values of MCI patients increased in the MTG, SPL, MFG, SMG, cuneus and PCG and decreased in the frontal regions (IFG, MFG and SFG), fusiform gyrus, PHIP, ITG and PCC. The results suggested that several regions of DMN showed different ReHo values between MCI patients and HC during the post acupuncture resting state. Different effective connectivity have been reported related with the DMN regions during the post acupuncture resting state in HC in the previous study about the acupuncture specificity [20]. Our results further indicated the effects of acupuncture on the DMN regions in MCI patients and HC. In addition, the results showed that acupuncture increased regional functional connectivity of the abnormal regions related to the disease in MCI patients, aiming to rehabilitate the function of the abnormal regions. In practice, the well-identified physical effects of acupuncture needling and its purported clinical efficacy also suggest that acupuncture acts in maintaining a homeostatic balance of the internal state [52]. We speculated that the different responses to acupuncture between MCI patients and healthy controls reflected the rebalance effects of acupuncture. The rebalance effects of acupuncture may be related to mechanisms of the effects of acupuncture on MCI patients.

There are still some limitations in the study. First, the experience of acupuncture may affect the acupuncture effects. In the present study, we just included acupuncture naïve subjects, the difference in its efficacy between acupuncture naive and experienced may be explored in the future. Second, no clinical outcomes were recorded in the present study. We did not take any cognitive measures in the study for the subjects only received one acupuncture trial. Cognitive measures should be taken in future longitude studies. Third, the sample size in this experiment is small. Although ReHo, the measure we selected in this study, is a widely used measure to characterize local functional homogeneity of resting state fMRI signals with its test-retest reliability established, we should perform further study with more subjects in the future.

## Conclusions

In conclusion, we revealed some features of neural responses to acupuncture for MCI patients compared with healthy controls. Firstly, we found several brain regions showing different ReHo values between MCI patients and healthy controls. Furthermore, we found significant ReHo changes related with the previous reported abnormal regions in MCI patients during the post acupuncture resting state. Finally, we explored the different responses to acupuncture in MCI patients and healthy controls. We found some ReHo changes during the post acupuncture resting state between MCI patients and healthy controls. However, the two-way ANCOVA results showed that none of the effects are significant after multiple corrections. The reason why the results showed no statistically significant acupuncture effect may be the small sample size. The results suggested that the modulatory effects of acupuncture on MCI patients should be further investigated with more subjects in the future.
